# The impact of 2023 EMA recommendations on patients treated with JAK inhibitors: real-life experience from a prospective monocentric cohort

**DOI:** 10.1093/rheumatology/kead395

**Published:** 2023-07-31

**Authors:** Alessandro Tomelleri, Giovanni Benanti, Adriana Cariddi, Stefania Laura Calvisi, Elena Baldissera, Nicola Boffini, Marco Matucci-Cerinic, Lorenzo Dagna

**Affiliations:** Unit of Immunology, Rheumatology, Allergy and Rare Diseases, IRCCS San Raffaele Hospital, Milan, Italy; Vita-Salute San Raffaele University, Milan, Italy; Unit of Immunology, Rheumatology, Allergy and Rare Diseases, IRCCS San Raffaele Hospital, Milan, Italy; Vita-Salute San Raffaele University, Milan, Italy; Unit of Immunology, Rheumatology, Allergy and Rare Diseases, IRCCS San Raffaele Hospital, Milan, Italy; Unit of Immunology, Rheumatology, Allergy and Rare Diseases, IRCCS San Raffaele Hospital, Milan, Italy; Unit of Immunology, Rheumatology, Allergy and Rare Diseases, IRCCS San Raffaele Hospital, Milan, Italy; Unit of Immunology, Rheumatology, Allergy and Rare Diseases, IRCCS San Raffaele Hospital, Milan, Italy; Unit of Immunology, Rheumatology, Allergy and Rare Diseases, IRCCS San Raffaele Hospital, Milan, Italy; Department of Experimental and Clinical Medicine, University of Florence, Florence, Italy; Unit of Immunology, Rheumatology, Allergy and Rare Diseases, IRCCS San Raffaele Hospital, Milan, Italy; Vita-Salute San Raffaele University, Milan, Italy

Dear Editor, The Janus kinase inhibitors (JAKis) are a promising class of drugs for treating immune-mediated diseases [1]. However, recent data suggest that JAKis may be associated with an increased risk of cardiovascular and thromboembolic events and malignancies [2, 3]. As a result, the European Medicines Agency (EMA) recommends that these drugs should be used in patients ≥65 years of age or those at increased risk of major cardiovascular events (MACE) and cancer only if no suitable alternatives are available [4]. Moreover, the EMA also suggests caution when using JAKis in patients with risk factors for venous thromboembolism (VTE) [5]. The EULAR advocates for shared decision making between the patient and rheumatologist when treating patients with arthritis [6].

This study prospectively evaluated the impact of the EMA recommendations on the management of patients with rheumatic diseases already on JAKi treatment at a tertiary Italian hospital. We enrolled all consecutive patients on JAKi with a scheduled follow-up visit between December 2022 and April 2023 and collected data on the patients’ age, previous therapies and history of/risk factors for MACE, VTE and cancer. All patients, regardless of age and comorbidities, discussed with the rheumatologist the new EMA recommendations and the opportunity of maintaining or discontinuing JAKi based on such recommendations. The final decision, the reason(s) for that decision and whether the decision was primarily led by the physician or patient were recorded. All patients provided consent for anonymous use of their demographic and clinical data.

We evaluated 223 patients with rheumatic diseases on JAKi (Supplementary Table S1, available at *Rheumatology* online, summarizes their main demographic and clinical characteristics). Following the visit, JAKi treatment was interrupted in 29 (13%) patients. In all cases, the patient and rheumatologist shared the decision, as advocated by the EULAR recommendations [6]. In 3 patients (10%), the decision was primarily led by the patient due to concerns regarding the risk of MACE or cancer. In 26 patients (90%), the rheumatologist was the first to propose a therapeutic modification. The medical history of patients who withdrew JAKi and reasons that led to the decision to withdraw JAKi are described in detail in Table 1. The mean age of patients who changed therapy was 66 years (s.d. 11) and 17 of them (59%) were ≥65 years of age. Nineteen patients (73%) had at least one cardiovascular risk factor. The most common cardiovascular risk factor among patients who changed treatment was dyslipidaemia [*n* = 16 (55%)], followed by smoking [*n* = 13 (45%)], hypertension [*n* = 12 (41%)], diabetes [*n* = 8 (28%)], obesity [*n* = 2 (7%)] and chronic coronary disease [*n* = 2 (7%)]. A previous diagnosis of cancer and deep vein thrombosis was present in 4 (14%) and 2 (7%) patients, respectively. No patient had a previous history of acute coronary syndrome or stroke/transient ischaemic attack. Among patients who suspended JAKi, in 12 cases (41%) this decision was primarily made because the drug was deemed primarily (75%) or secondarily (25%) ineffective by the rheumatologist (i.e. patients had moderate or high disease activity at clinical evaluation). Hence, among patients who were adequately responding to JAKi, only 17/211 (8%) changed the treatment. After JAKi withdrawal, patients were swapped to TNF inhibitors [*n* = 12 (41%)], tocilizumab [*n* = 8 (28%)], abatacept [*n* = 2 (7%)] or sarilumab [*n* = 1 (3%)]. Six patients (21%) were maintained on conventional synthetic DMARD monotherapy.

**Table 1.** Demographic features and clinical history of patients with rheumatic diseases who stopped a JAKi between December 2022 and April 2023

Patient	Sex, age	Disease, disease duration (months)	Previous bDMARDs, *n*	Type of JAKi, dose	Duration of JAKi therapy, months	Concomitant therapy	Cancer risk factors	CV risk factors	VTE risk factors	History of cancer	History of MACE	History of VTE	Disease status	Person who led the decision	New therapy
1	Male, 28	PsA, 59	3	UPA, 15 mg	6	–	No	No	No	No	No	No	MDA	Rheumatologist	CZP
2	Female, 74	RA, 147	1	BARI, 4 mg	42	MTX	No	Dyslipidaemia	No	No	No	No	Remission	Patient	ABA
3	Female, 64	RA, 323	1	FIL, 200 mg	6	MTX, PDN 1.25 mg	No	Dyslipidaemia, diabetes	No	No	No	No	MDA	Rheumatologist	ETN
4	Female, 56	RA, 139	6	UPA, 15 mg	31	MTX, PDN 5 mg	No	Hypertension	No	Melanoma	No	No	MDA	Rheumatologist	TCZ
5	Female, 67	PsA, 311	5	UPA, 15 mg	19	MTX, PDN 5 mg	Smoking	Hypertension, smoking	Smoking	No	No	No	Remission	Rheumatologist	MTX monotherapy
6	Male, 72	RA, 176	1	FIL, 200 mg	3	LEF, PDN 5 mg	Smoking	Dyslipidaemia, diabetes	Smoking	No	No	No	MDA	Rheumatologist	ETN
7	Female, 80	RA, 275	3	FIL, 200 mg	8	LEF	Smoking	No	No	No	No	No	LDA	Rheumatologist	ETN
8	Female, 56	RA, 92	1	BARI, 4 mg	43	–	No	Dyslipidaemia, hypertension, coronary disease	No	No	No	No	LDA	Rheumatologist	ETN
9	Female, 59	RA, 93	2	FIL, 200 mg	49	MTX	No	Dyslipidaemia	No	No	No	No	Remission	Patient	SARI
10	Female, 70	RA, 251	1	BARI, 4 mg	16	HCQ	Smoking	No	No	No	No	No	MDA	Rheumatologist	HCQ monotherapy
11	Female, 77	RA, 156	0	BARI, 2 mg	53	–	No	No	No	Thyroid cancer	No	No	Remission	Rheumatologist	LEF monotherapy
12	Female, 76	RA, 64	0	TOFA, 5 mg × 2	47	–	No	Dyslipidaemia, hypertension	No	No	No	No	LDA	Patient	TCZ
13	Male, 62	RA, 96	1	FIL, 200 mg	10	MTX, PDN 2.5 mg	Smoking	Smoking, obesity, dyslipidaemia, diabetes	Smoking, obesity	No	No	No	HDA	Rheumatologist	MTX monotherapy
14	Male, 74	RA, 181	6	FIL, 200 mg	48	MTX, PDN 2.5 mg	Smoking	Smoking, dyslipidaemia, diabetes, hypertension	Smoking	No	No	No	MDA	Rheumatologist	ETN
15	Female, 70	RA, 204	0	FIL, 100 mg	19	–	No	Hypertension	No	No	No	No	HDA	Rheumatologist	TCZ
16	Female, 80	RA, 92	0	BARI, 4 mg	58	–	No	No	No	Breast cancer	No	No	Remission	Rheumatologist	ETN
17	Female, 62	RA, 80	1	FIL, 200 mg	56	MTX	No	No	No	No	No	DVT	LDA	Rheumatologist	ETN
18	Female, 81	RA, 24	1	UPA, 15 mg	4	MTX	Smoking	Smoking, dyslipidaemia, hypertension, coronary disease	Smoking	No	No	No	LDA	Rheumatologist	ETN
19	Female, 85	RA, 97	0	TOFA, 5 mg × 2	54	MTX	–	Obesity, dyslipidaemia, hypertension	Obesity	No	No	No	Remission	Rheumatologist	MTX monotherapy
20	Female, 75	RA, 157	1	BARI, 4 mg	17	LEF	Smoking	Smoking, dyslipidaemia, hypertension	Smoking	Myelodysplastic syndrome	No	No	MDA	Rheumatologist	TCZ
21	Female, 81	RS3PE, 20	0	FIL, 100 mg	11	MTX	No	Diabetes	No	No	No	No	LDA	Rheumatologist	TCZ
22	Male, 67	RA, 109	2	BARI, 2 mg	60	MTX	Smoking	Smoking	Smoking	No	No	No	MDA	Rheumatologist	TCZ
23	Female, 57	RA, 158	2	FIL, 200 mg	49	SSZ	Smoking, family history	Smoking, dyslipidaemia, hypertension	Smoking	No	No	No	LDA	Rheumatologist	TCZ
24	Male, 66	RA, 55	0	UPA, 15	26	–	Smoking	Smoking, dyslipidaemia	Smoking	No	No	No	Remission	Rheumatologist	ETN
25	Female, 58	RA, 146	2	UPA, 15 mg	10	–	No	No	No	No	No	DVT	MDA	Rheumatologist	ADA
26	Female, 59	RA, 52	1	FIL, 200 mg	21	MTX	Smoking	Smoking, diabetes	Smoking	No	No	No	MDA	Rheumatologist	ABA
27	Female, 53	RA, 44	1	BARI, 2 mg	26	–	No	Dyslipidaemia, hypertension, diabetes	No	No	No	No	LDA	Rheumatologist	TCZ
28	Female, 72	RA, 133	0	BARI, 2 mg	54	MTX	No	Dyslipidaemia Hypertension Diabetes	No	No	No	No	LDA	Rheumatologist	MTX monotherapy
29	Female, 63	RA, 337	2	UPA, 15 mg	18	–	Smoking	Smoking, dyslipidaemia, hypertension	Smoking	Breast cancer	No	No	Remission	Rheumatologist	TCZ

Data from patients at our unit. Factors that guided final decision and subsequent management are also reported.

ABA: abatacept; ADA: adalimumab; BARI: baricitinib; bDMARDs: biologic DMARDs; csDMARDs: conventional synthetic DMARDs; CV: cardiovascular; CZP: certolizumab pegol; DVT: deep vein thrombosis; ETN: etanercept; FIL: filgotinib; HCQ, hydroxychloroquine; HDA: high disease activity; LDA: low disease activity; MDA: moderate disease activity; PDN: prednisone; RS3PE: remitting seronegative symmetrical synovitis with pitting oedema; SARI: sarilumab; TCZ: tocilizumab; TOFA: tofacitinib; UPA: upadacitinib.

In conclusion, our data show that only a very small proportion of patients with arthritis treated with JAKi requested therapy discontinuation due to concerns about the recent EMA recommendations [3]. This testifies to a general confidence of patients to continue receiving JAKi if they are provided with adequate information. In most cases, the rheumatologists themselves proposed the treatment modification due to various factors, mostly the patients’ age and the presence of cardiovascular risk factors. Moreover, in nearly half of patients who suspended JAKi, a lack of efficacy (mostly secondary inefficacy) was the reason that led to the final decision. On the other hand, it is worth noting that, in our cohort, JAKi therapy ensured optimal disease control overall, as evidenced by the relatively low fraction of patients with moderate/high disease activity (5%).

The strengths of this study are its prospective nature, the large number of patients included from a monocentric cohort and their demographic heterogeneity. The main shortcoming is the absence of data on the outcome of patients who withdrew JAKi. A comparison of our findings with those from other cohorts to further confirm these data is certainly warranted.

## Supplementary Material

kead395_Supplementary_Data

## Data Availability

The data that support the findings of this study are available upon request from the corresponding author.
