# Supplementary material for: The impact of 2023 EMA recommendations on patients treated with JAK inhibitors: real-life experience from a prospective monocentric cohort
Source: Rheumatology (Oxford). 2023 Jul 31;63(2):e39–42. doi: 10.1093/rheumatology/kead395 (PMC10836993; doi:10.1093/rheumatology/kead395)
Supplement: kead395_Supplementary_Data [file kead395_supplementary_data.docx]

| Female sex, n (%) | 163 (73) |
| --- | --- |
| Age (years), mean ± SD | 60 ± 12.8 |
| Disease, n (%) |  |
| Rheumatoid arthritis | 210 (94) |
| Psoriatic arthritis | 11 (5) |
| Polymyalgia rheumatica/RS3PE | 1 (0.5) |
| Juvenile idiopathic arthritis | 1 (0.5) |
| Disease duration (months), mean ± SD | 142 ± 106.2 |
| Previous treatment with ≥1 bDMARD(s), n (%) | 123 (55) |
| JAK-inhibitor, n (%) |  |
| Baricitinib | 55 (25) |
| Filgotinib | 78 (35) |
| Tofacitinib | 20 (9) |
| Upadacitinib | 70 (31) |
| Duration of JAK-i therapy (months), mean ± SD | 30.1 ± 18.3 |
| Concomitant glucocorticoids, n (%) | 34 (15) |
| Prednisone dose, mg daily, mean ± SD | 5 ± 2.3 |
| Concomitant csDMARD, n (%) | 102 (46) |
| Methotrexate | 64 (29) |
| Leflunomide | 24 (11) |
| Sulfasalazine | 1 (0.5) |
| Hydroxychloroquine | 13 (6) |
| Number of previous bDMARDs, mean ± SD | 2 ± 1.3 |
| Risk Factors, n (%) |  |
| Age ≥ 65 years | 79 (35) |
| Smoking | 73 (33) |
| Arterial hypertension | 64 (29) |
| Hyperlipidaemia | 81 (36) |
| Obesity | 30 (13) |
| Diabetes mellitus | 24 (11) |
| Coronary disease | 5 (2) |
| History of MACE, n (%) |  |
| Acute coronary syndrome | 1 (0.5) |
| Stroke/TIA | 1 (0.5) |
| Venous thromboembolism | 3 (1) |
| History of cancer, n (%) | 12 (5) |

**Supplementary Table S1.** Demographic and clinical features of patients with a rheumatic disease and on JAK-i therapy who attended our Unit between December 2022 and April 2023.

bDMARDs, biological disease modifying anti-rheumatic drugs; csDMARDs conventional synthetic disease modifying anti-rheumatic drugs; JAK, janus kinase; MACE, major adverse cardiovascular events; RS3PE, remitting seronegative symmetrical synovitis with pitting oedema; SD, standard deviation; TIA, transient ischemic attack.
